# Liver cirrhosis: current status and treatment options using western or traditional Chinese medicine

**DOI:** 10.3389/fphar.2024.1381476

**Published:** 2024-07-16

**Authors:** Shihao Zheng, Chengyuan Xue, Size Li, Xiaobin Zao, Xiaoke Li, Qiyao Liu, Xu Cao, Wei Wang, Wenying Qi, Hongbo Du, Peng Zhang, Yongan Ye

**Affiliations:** ^1^ Dongzhimen Hospital, Beijing University of Chinese Medicine, Beijing, China; ^2^ Beijing University of Chinese Medicine, Beijing, China; ^3^ Key Laboratory of Chinese Internal Medicine of Ministry of Education and Beijing, Dongzhimen Hospital, Beijing University of Chinese Medicine, Beijing, China; ^4^ Liver Diseases Academy of Traditional Chinese Medicine, Beijing University of Chinese Medicine, Beijing, China; ^5^ Dongfang Hospital, Beijing University of Chinese Medicine, Beijing, China

**Keywords:** liver cirrhosis, current status, treatment options, Chinese medicine, pharmacology

## Abstract

Liver cirrhosis arises from liver fibrosis and necroinflammation caused by various mechanisms of hepatic injury. It is a prevalent condition in clinical practice characterized by hepatocellular dysfunction, portal hypertension, and associated complications. Despite its common occurrence, the etiology and pathogenesis of liver cirrhosis remain incompletely understood, posing a significant health threat. Effective prevention of its onset and progression is paramount in medical research. Symptoms often include discomfort in the liver area, while complications such as sarcopenia, hepatic encephalopathy, ascites, upper gastrointestinal bleeding, and infection can arise. While the efficacy of Western medicine in treating liver cirrhosis is uncertain, Chinese medicine offers distinct advantages. This review explores advancements in liver cirrhosis treatment encompassing non-pharmacological and pharmacological modalities. Chinese medicine interventions, including Chinese medicine decoctions, Chinese patent medicines, and acupuncture, exhibit notable efficacy in cirrhosis reversal and offer improved prognoses. Nowadays, the combination of Chinese and Western medicine in the treatment of liver cirrhosis also has considerable advantages, which is worthy of further research and clinical promotion. Standardized treatment protocols based on these findings hold significant clinical implications.

## Epidemiology of liver cirrhosis

**FIGURE 1:**
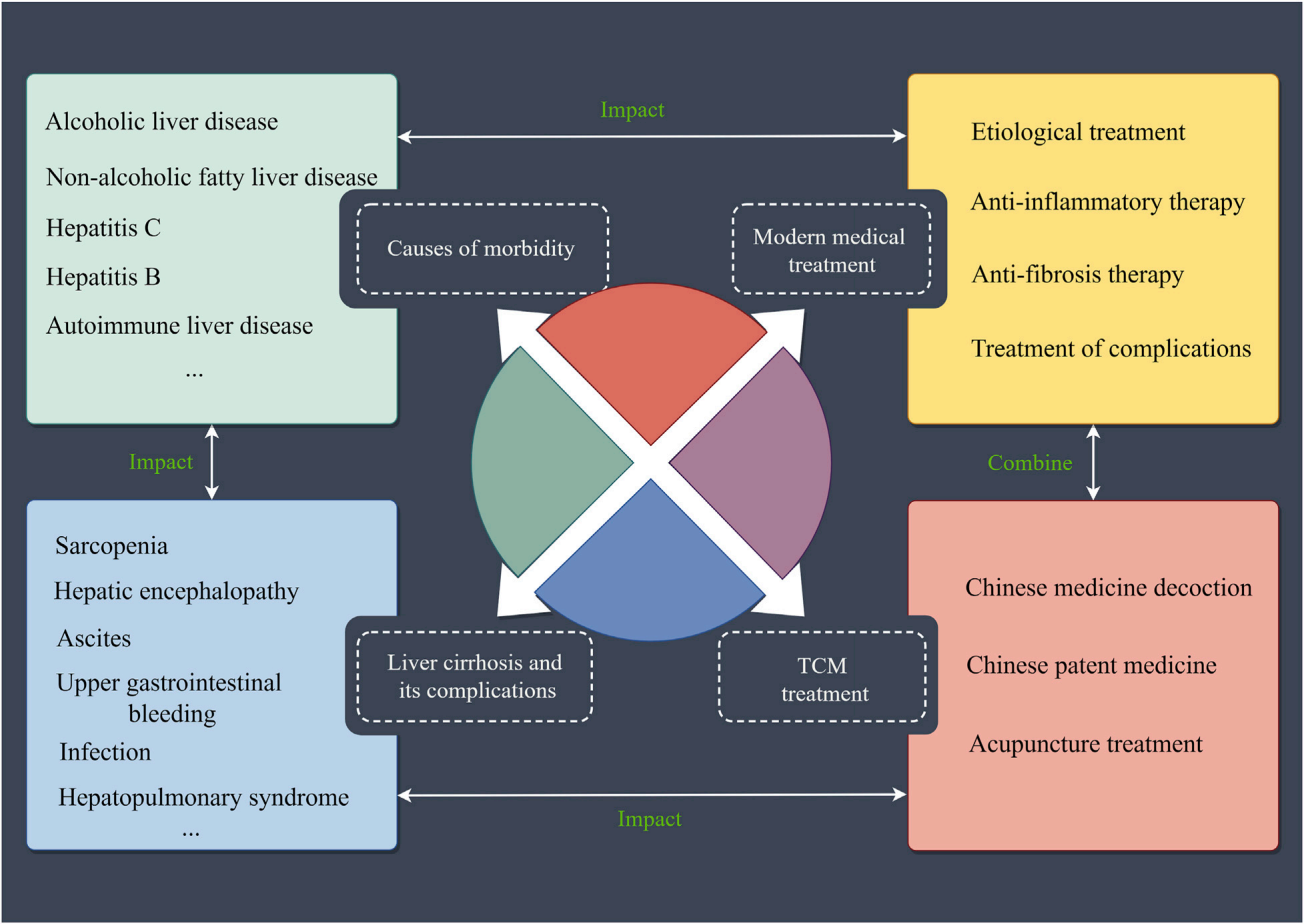
Liver cirrhosis and its associated factors. Mainly includes the causes of morbidity and complications of liver cirrhosis, as well as modern medical and Chinese medicine treatments for liver cirrhosis. TCM: Traditional Chinese Medicine.

Liver cirrhosis is the result of fibrosis and necrotizing inflammation stemming from various mechanisms of liver injury (Figure 1). Histologically, the condition is characterized by the presence of regenerated nodules encased in dense fibrous septa, leading to the disappearance of parenchyma and subsequent collapse of liver structure (Schuppan and Afdhal, 2008; Ginès et al., 2021). This process ultimately results in the distortion and deformation of the liver’s vascular architecture. The term “cirrhosis” originates from the Greek word “kirrhos,” denoting a yellow or yellowish-brown hue, in reference to the coloration of nodules visible on the liver’s surface.Today, liver cirrhosis represents the advanced pathological progression of chronic liver disease, characterized by persistent inflammation, restructuring of hepatic lobes, and the development of pseudolobules and nodules (Nie et al., 2023). And Liver cirrhosis it can present clinically in two main stages: an asymptomatic stage known as compensated cirrhosis, and a symptomatic stage known as decompensated cirrhosis. In the latter stage, the primary manifestations, including jaundice, ascites, gastrointestinal bleeding, and encephalopathy, pose significant risks to human health (Costa et al., 2022).

Liver cirrhosis, a significant component of digestive diseases, presents a substantial economic burden in numerous countries. However, the scarcity of data regarding the incidence and mortality rates of liver cirrhosis in regions with high prevalence, notably Africa, has garnered considerable attention from scholars globally. The prevalence of liver cirrhosis is estimated to be between 4.5% and 9.5% of the global population (Starr and Raines, 2011; Llovet et al., 2016; Fateen and Ryder, 2017). Annually, approximately two million deaths worldwide are attributed to liver diseases, with one million resulting from cirrhosis and another one million due to viral hepatitis and hepatocellular carcinoma (HCC) (GBD, 2017 Cirrhosis Collaborators, 2020). Liver cirrhosis is the 11th most common cause of death globally and the third leading cause of death among individuals aged 45–64 years. When combined with liver malignancies, cirrhosis accounts for 3.5% of all deaths worldwide (Asrani et al., 2019). A major study found that approximately 2.2 million adults in the U.S. have cirrhosis, and that the annual death rate from cirrhosis rose from 14.9 per 100,000 to 21.9 per 100,000 in the decade to 2021, placing a serious healthcare burden on U.S. society, as well as a significant economic impact globally (Tapper and Parikh, 2023; Wazir et al., 2023). Surprisingly, deaths due to cirrhosis already accounted for 2.4% of global deaths in 2019, and the number of people dying from cirrhosis is expected to continue to increase over the next decade (Huang et al., 2023). However, it is important to note that data on mortality rates related to chronic liver diseases, such as cirrhosis, may be conservative, and research studies may not fully capture the true extent of the global burden (Asrani et al., 2013). The treatment of liver diseases such as liver cirrhosis is an onerous and arduous task, waiting for further exploration.

## Understanding liver cirrhosis in modern medicine

### Etiology and pathogenesis of liver cirrhosis

The causes of cirrhosis are diverse, with most chronic liver diseases potentially leading to liver fibrosis and eventual cirrhosis. Common causes include alcoholic liver disease, non-alcoholic fatty liver disease, hepatitis C, and hepatitis B. In most Asian countries, chronic hepatitis B infection predominates as the main cause of cirrhosis, while in European and American nations, hepatitis C, alcoholic, or non-alcoholic fatty liver diseases are primary contributors. Viral hepatitis, specifically hepatitis B and hepatitis C, often underlie liver fibrosis or cirrhosis, while hepatitis A and hepatitis E typically manifest as acute self-limiting hepatitis. Chronic hepatitis E is rare and mainly occurs in individuals with immune deficiencies or those using immunosuppressive drugs, akin to non-hepatotropic hepatitis mechanisms such as Epstein-Barr virus (Liu et al., 2024) and cytomegalovirus (Heusel et al., 2024). Some scholars have proposed autoimmune mechanisms, chronic liver diseases, schistosomiasis, and malnutrition as potential cirrhosis causes, yet these remain contentious and necessitate further investigation (Bartolotta et al., 2022). However, from the perspective of Chinese medicine, various pathogenic factors can lead to the emergence of pathological factors such as qi stagnation, dampness-heat, blood stasis, etc., which injure the liver and ultimately lead to the onset and development of cirrhosis.

**FIGURE 2:**
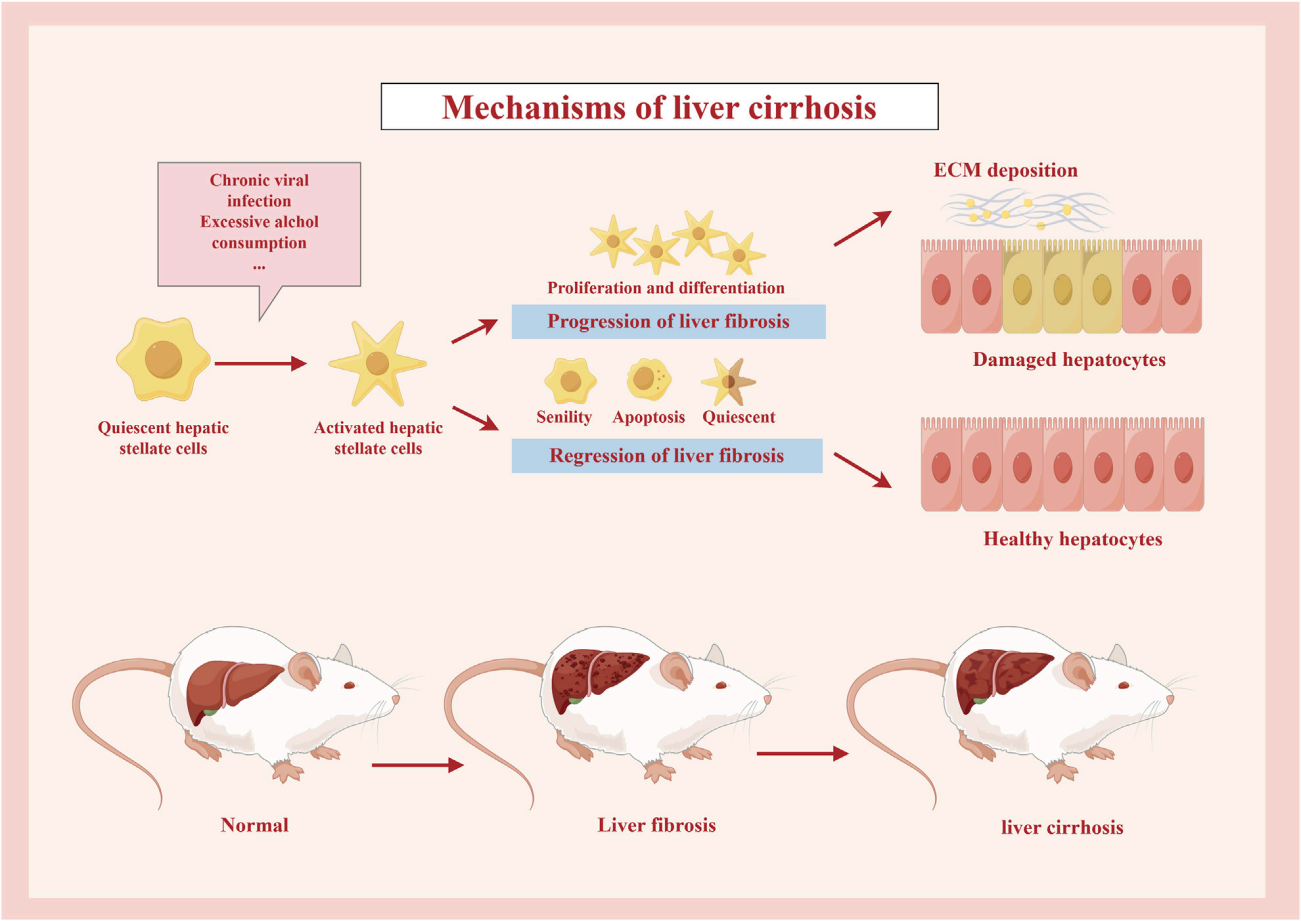
Mechanisms of liver cirrhosis. Under the stimulation of the relevant etiological factors, the quiescent hepatic stellate cells are activated, and some of them proliferate and differentiate, eventually forming ECM deposits, leading to the development of liver fibrosis and then cirrhosis, while some other cells are restored to healthy hepatocytes by external interventions or their own causes, etc.

Liver cirrhosis represents a post-injury liver repair response wherein hepatic stellate cell (HSC) activation plays a pivotal role, alongside liver sinusoidal endothelial cells (LSECs) regulation, cytokine release, and fibrous scar tissue deposition (Kisseleva and Brenner, 2021). Cirrhosis typically progresses from chronic hepatitis to liver fibrosis and ultimately cirrhosis (Romanelli and Stasi, 2016) (Figure 2). Following liver injury from various pathological factors, macrophages, T cells, and other inflammatory cells secrete numerous chemokines and inflammatory factors, instigating immune cell recruitment to the injury site. This cascade activates the liver’s inflammatory response, causing hepatocyte inflammation, damage, and potentially necrosis and apoptosis (Campana and Iredale, 2017; Krenkel et al., 2018; Engelmann et al., 2023). Within this inflammatory milieu, HSCs transition into myofibroblasts, prompting excessive extracellular matrix (ECM) production, and angiogenic factor release (Hernandez-Gea and Friedman, 2011; Roehlen et al., 2020). Research suggests that HSCs typically constitute 5%–8% of a healthy human liver, primarily regulating ECM synthesis and degradation to modulate hepatic sinusoidal blood supply (El-Serag, 2012).

LSECs (Gracia-Sancho et al., 2021; McConnell et al., 2023) serve as vital immune barriers within the liver, safeguarding against foreign body recognition, phagocytosis, and degradation. However, under inflammatory conditions, LSECs lose their specialized phenotype, leading to pore loss. Damaged LSECs secrete pro-inflammatory and pro-fibrotic factors, activating HSCs and promoting ECM deposition between hepatic sinusoidal spaces. This process forms fibrous septa with vascular proliferation, culminating in hepatic fibrosis progression. Continued fibrosis leads to septal extension, pseudolobule formation, and eventual liver parenchyma destruction and vascular structure distortion, resulting in cirrhosis (Ni et al., 2017; Li, 2022; Gao et al., 2024). Autophagy, a mechanism involved in liver injury, influences disease development (Kouroumalis et al., 2021). Early-stage autophagy protects LSECs, maintaining endothelial cell phenotype and liver cell redox balance to prevent oxidative stress. Loss of endothelial autophagy exacerbates endothelial dysfunction, impacting liver fibrosis by specifically regulating HSC activation.

A healthy liver performs various functions, including metabolism, detoxification, nutrient storage, and bile production for digestion (Mancak et al., 2024). Triggers such as hepatic fat accumulation, drug-induced injury, excessive alcohol consumption, or viral hepatitis can induce liver inflammation and fibrosis. Advanced fibrosis and substantial liver structure damage lead to cirrhosis, eventually predisposing individuals to liver cancer (Toh et al., 2023). Regular medical check-ups are advisable, especially for individuals with chronic liver diseases, family history, or other risk factors, as early detection and treatment are crucial in combating end-stage liver disease (Yang et al., 2019).

### Liver cirrhosis and its complications

#### Liver cirrhosis and sarcopenia

Sarcopenia represents a prevalent complication in individuals with liver disease, with mounting evidence indicating its significant prognostic implications for cirrhosis patients. Ponziani and Gasbarrini (2018) reported that sarcopenia afflicts approximately 70% of individuals with end-stage liver disease. Compared to numerous chronic conditions, sarcopenia’s occurrence in cirrhosis patients is notably high. While its prevalence in patients with cardiovascular diseases stands at 29.7%, those with liver cirrhosis experience a strikingly elevated rate of 48.1% (Kim et al., 2017). Research by Ponziani and Gasbarrini (2018) found sarcopenia incidence among cirrhosis patients to fluctuate between 20% and 70%. Additionally, a study by Hanai et al. (2016) observed cirrhosis patients exhibiting over twice the muscle loss rate compared to healthy individuals, with a notable increase in mortality when muscle loss exceeded 3%. Presently, burgeoning research delves into cirrhosis and sarcopenia, with findings indicating potential pathogenic links to systemic inflammatory responses (Keller et al., 2011; Sinclair et al., 2016), heightened catabolic states (Plauth et al., 2019), liver-muscle interactions (Qiu et al., 2013; Welch et al., 2020), inadequate energy intake (Cheung et al., 2012), and disruptions in protein homeostasis (Periyalwar and Dasarathy, 2012; Tandon et al., 2021).

#### Liver cirrhosis and hepatic encephalopathy

Hepatic encephalopathy stands as a prevalent complication of cirrhosis and ranks among the primary causes of mortality in affected patients. It manifests as a metabolic disorder stemming from central nervous system dysfunction in individuals with acute or chronic liver ailments. Nevertheless, its occurrence in cirrhosis patients signifies heightened mortality risk and a relatively grim prognosis (Shen et al., 2015). Presently, the precise pathogenesis of hepatic encephalopathy remains incompletely elucidated, with theories encompassing ammonia toxicity, neurotransmitter imbalances, and amino acid perturbations, among which ammonia toxicity serves as a focal point. Risk factors for hepatic encephalopathy in cirrhosis patients encompass infections, ascites, electrolyte imbalances, and upper gastrointestinal bleeding. Furthermore, Maqsood et al. (2006) identified prothrombin time as a prominent precipitating factor for hepatic encephalopathy. Additionally, hyponatremia-induced reduction in extracellular osmotic pressure and resultant cerebral edema are deemed significant contributors to hepatic encephalopathy development, with a reported 8% increase in hepatic encephalopathy risk for every 1 mmol/L reduction in serum sodium (Iwasa and Takei, 2015; Bossen et al., 2019). Addressing the substantial incidence of hepatic encephalopathy in cirrhosis patients warrants profound attention and contemplation for future interventions.

#### Liver cirrhosis and ascites

Ascites represent a prevalent complication of cirrhosis and stand as a common ailment encountered in clinical practice (Ginés et al., 1987; Runyon et al., 1992). Its emergence often signals an advanced stage of cirrhosis, with a prognosis characterized by complexity and variability. Particularly in refractory cirrhosis, ascites often accompany other cirrhosis-related complications, leading to a poor clinical outlook and a significant decline in patients’ quality of life. Cirrhotic ascites refers to the accumulation of free fluid within the abdominal cavity of cirrhosis patients. Substantial ascitic fluid can induce symptoms like reduced appetite and abdominal distension, profoundly impacting patients’ quality of life and heightening the risk of complications such as hepatic encephalopathy, hepatorenal syndrome, and spontaneous peritonitis (Tandon and Garcia-Tsao, 2008). The 1-year mortality rate among cirrhosis patients with ascites reaches 15%, while the 5-year mortality rate can soar as high as 44% (Biecker, 2011). The mechanism underlying ascite formation involves various factors, including portal hypertension, hypoalbuminemia, inadequate effective circulating blood volume, and heightened production of hepatic lymph fluid, all contributing to ascite development.

#### Liver cirrhosis and upper gastrointestinal bleeding

Liver cirrhosis is often accompanied by multiple system involvement, and complications such as upper gastrointestinal bleeding and secondary infection often occur in the late stage. Upper gastrointestinal bleeding is the most common fatal complication, and the overall mortality rate of upper gastrointestinal bleeding can reach 30 percent (de Franchis and Baveno VI Faculty, 2015; Dango et al., 2017). The risk of portal hypertension and upper gastrointestinal bleeding can be determined by detecting the pressure gradient of hepatic veins. It is worth noting that this examination is a traumatic examination, so electronic endoscopy, a non-traumatic examination, is often used clinically to evaluate the degree of esophageal varices in patients with cirrhosis, so as to prevent the occurrence of upper gastrointestinal bleeding (Song and Kim, 2019). Related studies have confirmed that patients with advanced cirrhosis are more likely to develop esophageal varices. The severity of liver disease is correlated with the grade of esophageal varices. About 45% of patients with Child-PughA cirrhosis have varicose veins (Elsebaey et al., 2019), while 85% of patients with Child-PughC cirrhosis have varicose veins (Sousa et al., 2017). Patients with cirrhosis of the liver have a higher incidence of upper gastrointestinal bleeding if they have bad lifestyle habits such as smoking and drinking.

#### Liver cirrhosis and infection

Patients with cirrhosis are prone to co-infection, which is one of the common causes of death in patients with cirrhosis. Related studies have confirmed that 39.7% of patients with cirrhosis can be complicated by various infections (Fernández et al., 2019). Piano et al. (2021) found through their research that the infection rate of hospitalized patients with cirrhosis was high, which was 4–5 times that of normal population, and the infection rate of patients with decompensated cirrhosis was up to 67%. Liver cirrhosis complicated with infection is also prone to septic shock, which can lead to multiple organ failure, leading to liver dysfunction, liver cell damage, and eventually liver failure and death (Hsu et al., 2021). Multiple mechanisms are involved in the co-infection of cirrhosis. First of all, the liver is closely related to the body’s immunity. Patients with cirrhosis have low liver reserve function, and their innate and acquired immune defense capabilities as well as the ability to remove microorganisms and toxins are severely impaired. Clinically, cirrhosis may be characterized by increased susceptibility to infection and poor prognosis after complicated with sepsis (Lebossé et al., 2019; Basho et al., 2021). Secondly, the characteristic clinical manifestation of decompensation in cirrhosis is portal hypertension. Anatomically, blood flow from the internal organs and intestines is connected to the portal vein, which enables bacteria in the intestine to enter the systemic circulation blood system through the collateral branches of the portal vein circulation and cause systemic infection (Tripathi et al., 2018; Solé et al., 2021). Another possibility is that portal hypertension in cirrhosis may show the characteristics of spleen enlargement in the early stage, and the occurrence of spleen enlargement may be accompanied by hypersplenism, which will further affect the immune function of the body, thus aggravating the development of liver cirrhosis (Asanoma et al., 2014).

#### Liver cirrhosis and hepatopulmonary syndrome

Hepatopulsyndrome (HPS), first proposed by Knudson and Kennedy in 1977 (Kennedy and Knudson, 1977), is a clinical syndrome characterized by arterial oxygenation dysfunction due to dilation of blood vessels in the lung, characterized by dyspnea in upright position and dyspnea after movement (Weinfurtner and Forde, 2020). HPS is most common in patients with cirrhosis, and can also be seen in acute and chronic hepatitis, acute liver failure, etc., which is one of the common complications of chronic liver disease (Machicao et al., 2014). One study (Fallon et al., 2008) found that non-HPS patients with a similar degree of cirrhosis were only half as likely to die as those with HPS. Epidemiological studies have also shown that the incidence of HPS in adults with end-stage liver diseases such as cirrhosis ranges from 4% to 47% (Varghese et al., 2007), while the prevalence of HPS in all patients with cirrhosis is 20% (Møller et al., 2009). The prevalence of HPS in patients with cirrhosis requiring liver transplantation is about 5%–30% of the population (Rodríguez-Roisin and Krowka, 2008). Modern medicine has also found that the pathogenesis of HPS may be related to the release of various cytokines in the body resulting in angiogenesis and pulmonary vasodilation caused by cirrhosis. As a serious pulmonary complication of end-stage liver disease, HPS seriously affects the quality of life and prognosis of patients. When dyspnea and hypoxemia occur in patients with chronic liver disease or portal hypertension, attention should be paid to screening whether HPS is combined, so as to achieve early detection, diagnosis and treatment to prevent deterioration of the condition.

#### Liver cirrhosis and hepatorenal syndrome

Hepatorenal syndrome (HRS) is a serious complication mainly manifested by renal injury in patients with severe liver diseases such as cirrhosis combined with ascites (Meola et al., 2016). Nowadays, with the deepening of HRS research, great progress has been made in pathogenesis and treatment. The International Club of Ascites (ICA) proposed the concept of acute kidney injury (AKI). On this basis, the diagnostic criteria and treatment guidelines of the HRS were revised (Angeli et al., 2015). Nowadays, with the deepening of HRS research, great progress has been made in pathogenesis and treatment. Studies (Al-Khafaji et al., 2015) have shown that 35%–40% of patients with cirrhosis combined with ascites may develop HRS. The main risk factors of HRS include portal hypertension, sepsis caused by spontaneous peritonitis, and cardiac insufficiency is also a high risk factor for HRS (Lenz et al., 2014). At present, global scholars have not fully understood the pathogenesis of HRS. Existing studies have found that due to the influence of end-stage liver diseases such as cirrhosis, the inactivation of vasodilator factors *in vivo* is reduced, which leads to the dilation of visceral and peripheral arteries, the contraction of renal vessels, the reduction of glomerular filtration rate and the decrease of renal blood flow, and finally the occurrence of HRS (Sobhonslidsuk, 2015; Simonetto et al., 2020; Wu et al., 2024). At the same time, liver inflammation, intestinal flora displacement and concurrent infection caused by cirrhosis lead to the release of a large number of inflammatory factors and endotoxemia, causing renal microcirculation dysfunction, which can induce renal function injury (Rad et al., 2024).

#### Liver cirrhosis and primary liver cancer

Primary liver cancer, as a high incidence of human malignant tumor, lacks typical clinical symptoms in the early stage, but the disease progresses rapidly, has a high risk of death, and the prognosis of patients is generally poor. Liver cirrhosis is a major risk factor for liver cancer and hepatology-related death. Therefore, accurate evaluation of the severity of cirrhosis is of great significance for clinical monitoring efficacy and prognosis assessment (Ren et al., 2017). Modern medical research has found that patients with cirrhosis with a genetic history of primary liver cancer have a significantly increased risk of developing primary liver cancer. Previous studies have found that the incidence of cirrhosis complicated with liver cancer is 1.5–6 percent per year (Bruix et al., 2011), and this figure may be increasing. Therefore, the prevention of primary liver cancer is urgent. More studies have found that long-term excessive drinking can increase the risk of liver cirrhosis complicated with primary liver cancer, especially after hepatitis B virus attacks the human liver, alcohol will cause increased liver burden, resulting in liver damage, and ultimately accelerate the occurrence and development of liver cancer. While avoiding excessive drinking for a long time, all countries should also strengthen health education about liver cirrhosis and primary liver cancer, improve patients’ further understanding of liver cirrhosis and primary liver cancer, enhance patients’ awareness of prevention and treatment, and reduce the incidence of liver cirrhosis complicated with primary liver cancer.

Research on liver cirrhosis and its complications underscores the significant impact of these conditions on patient prognosis and quality of life. While there are advancements in understanding risk factors and pathogenesis, challenges remain in the form of high variability in incidence rates, limited understanding of mechanisms, and the need for more effective management and preventive strategies. Further research is essential to address these gaps and improve patient outcomes.

## Treatment of liver cirrhosis using modern medicine

### Non-drug treatment

First of all, cirrhosis patients should maintain a reasonable diet. One study (Yao et al., 2018) found that the development of cirrhosis is associated with significant nutritional risks, often resulting in poor survival and serious complications. Dietary therapy is an important component of the treatment regimen for cirrhosis (Iwasa et al., 2013). Malnutrition affects 25 to 56 percent of patients with cirrhosis (Guglielmi et al., 2005; Huynh et al., 2015) and 65 to 90 percent of patients with advanced cirrhosis (Henkel and Buchman, 2006). Therefore, patients with cirrhosis should receive early nutritional interventions high in energy, carbohydrates, and protein, and eat multiple meals daily to ensure adequate nutrition.

Secondly, patients with cirrhosis should also maintain reasonable living habits, such as healthy work and rest time, and maintain a good mental state. Adequate sleep is essential for people, and studies (Marjot et al., 2021) have also found that problems such as lack of sleep are closely related to the pathogenesis of chronic liver disease. In conclusion, good living habits also play a very important role in the treatment and prognosis of cirrhosis.

Exercise therapy for liver cirrhosis has also received extensive attention from scholars. Exercise rehabilitation therapy is one of the important means of chronic disease conditioning. The American Association for the Study of Liver Disease recommends individualized exercise rehabilitation for patients with cirrhosis (Chen and Dunn, 2018). Another study (Ney et al., 2017) found that exercise levels were generally low in patients with cirrhosis, with 76% spending most of their time in a sitting position, which is detrimental to recovery from liver disease. In daily life, proper scientific exercises such as Tai Chi, five animal boxing/exercise, and eight trigrams boxing can effectively improve heart and lung function and prevent the occurrence of alcoholic fatty liver disease and cirrhosis.

Non-drug treatments for cirrhosis, including dietary therapy, healthy living habits, and exercise, offer significant benefits for patient outcomes. Proper nutrition and individualized exercise programs are crucial, though adherence to these regimens can be challenging. Further support and personalized plans are essential to maximize the effectiveness of these non-drug treatments.

### Drug treatment

After the diagnosis of liver cirrhosis is clear, comprehensive treatment should be started as soon as possible. In the treatment of cirrhosis, first attention should be paid to the etiological treatment, anti-inflammatory and anti-hepatic fibrosis treatment if necessary, and active prevention and treatment of complications. i) The key to the treatment of cirrhosis is etiological treatment. For example, all patients with cirrhosis who are positive for viral surface antigen should be evaluated for antiviral therapy, and patients should be treated with antiviral drugs, with the aim of delaying the progression of cirrhosis and reducing the incidence of liver cancer by inhibiting the replication of the virus (Manolakopoulos et al., 2009). Today, antiviral drugs include interferon, nucleoside and nucleotide drugs, such as Entecavir and tenofovir. One study (Marcellin et al., 2013) found that in 96 patients with HBV-associated cirrhosis followed up for 5 years after treatment with tenofovir dipivoxil, cirrhosis reversed in 71 patients, with a cure rate of 74 percent. It has also been found that treatment with standard antiviral drugs significantly increases the rate of sustained virological response and significantly reduces the recurrence rate of HCV (Morgan et al., 2010). In addition, a related antiviral agent, Adefovir dipivoxyl, has been found to be the antiviral strategy of choice for the treatment of hepatitis B-induced decompensated cirrhosis (Zhang et al., 2017). And there is also a prospective new study (Jagst et al., 2024) that found that hepatitis E tends to convert to chronic infection in immunocompromised patients, and that sofosbuvir is effective in inhibiting hepatitis E virus replication, especially in extrahepatic neuronal cells. ii) Anti-inflammatory and anti-fibrosis therapy should be considered in some patients with cirrhosis for whom etiological therapy is not available, or for whom liver inflammation or fibrosis remains severe after etiological therapy. Commonly used liver protection anti-inflammatory drugs are silymarin, glycyrrhizic acid preparations, polyene phosphatidylcholine, adenosine methionine, dicycloalcohol, reduced glutathione, ursodeoxycholic acid (UDCA) and so on. These hepatoprotective and anti-inflammatory drugs can inhibit liver inflammation, detoxify, regulate energy metabolism, remove reactive oxygen species and free radicals, improve the stability of liver cell membrane, immune regulation, integrity and fluidity, and ultimately achieve the purpose of reducing liver damage, reducing intrahepatic cholestasis, and promoting liver cell repair and regeneration (Damiris et al., 2020; Guo and Lu, 2020). Apart from this, the main antifibrotic drugs are Losartan (Gu et al., 2023) and Hydronidone. Recent studies (Sun et al., 2024) have also found that Hydronidone is able to induce apoptosis in activated HSC through the mitochondrial apoptotic pathway. iii) For the prevention and treatment of complications of liver cirrhosis, such as ascites, hepatic encephalopathy, etc. First-line ascites treatment includes: limiting salt intake and rational application of furosemid, spironolactone and other diuretics. A recent case report found that the optimal diuretic regimen for patients with hepatic pleural effusions may be furosemide in combination with spironolactone, yet this regimen was ineffective in about one-quarter of patients, requiring treatment such as thoracentesis (Malick et al., 2023). Second-line treatment includes rational application of vasoconstricting active drugs and other diuretics, such as tovaptan (Pratama et al., 2024) and terivasopressin (Ding et al., 2024); third-line treatment includes renal replacement therapy, ascites concentrated transfusion and liver transplantation. For the treatment of hepatic encephalopathy, the main treatment methods should be to correct the imbalance of amino acids, reduce the production of ammonia, promote the discharge of ammonia, reduce the absorption of enterotoxin, and maintain intestinal cleanliness. Also, rifaximin and lactulose/lactitol can be selected as the first line of treatment (Rajpurohit et al., 2022).

Drug treatments for liver cirrhosis, including etiological, anti-inflammatory, and anti-fibrosis therapies, as well as treatments for complications, offer significant benefits in managing the disease and improving patient outcomes. However, limitations such as variable patient responses, potential side effects, and the need for individualized treatment plans highlight the ongoing challenges in treating liver cirrhosis effectively. Further research and development of new therapies are needed to address these challenges and improve the long-term prognosis for patients with cirrhosis.

## Treatment of liver cirrhosis in traditional Chinese medicine

### Chinese medicine decoction

#### Yinchenhao decoction

Yinchenhao decoction (YCHD) is a Chinese medicine widely used in clinics to treat cirrhosis. Jiang et al. (2015) found that YCHD could significantly reverse changes in the expression of immunoregulatory genes in liver tissue, increase serum albumin level, effectively reduce the activity of serum alanine amino transportase, and reduce the degree of liver inflammation and necrosis. These changes were related to the regulation of the expression of genes related to immune/inflammatory response. Additionally, YCHD did not cause spleen weight loss or hepatocyte degeneration in mice. Cai et al. (2019) identified 45 key active ingredients and 296 potential therapeutic targets for YCHD anti-liver fibrosis through transcriptomic and network pharmacological analysis, indicating that YCHD mainly acts on apoptosation-related signaling pathways. *In vivo* experiments confirmed that YCHD treatment reduced the apoptosis of liver parenchymal cells and alleviated the symptoms of liver fibrosis. Further research by Cai et al. (2018) demonstrated that YCHD inhibits the activation and proliferation of HSCs through the TGF-β1/Smad/ERK signaling pathway. Liu et al. (2008) conducted a controlled experimental study on a variety of traditional prescriptions for cirrhosis and found that YCHD most significantly inhibited α-SMA activation and regulated rat cirrhosis. A systematic review and meta-analysis by Shi et al. (2021) provided high evidence that YCHD has a favorable modulatory effect on cholestasis, delaying the onset and progression of cirrhosis.

#### Xiaochaihu decoction

Xiaochaihu Decoction (XCHD), derived from Shanghan Zabing Lun, is a multi-functional classic decoction for treating a variety of diseases. Qiang et al. (2022) identified 164 key compounds of XCHD and 95 core targets of regulating liver fibrosis. They found that XCHD prevents liver cirrhosis by regulating several key pathways such as the TNF, IL-17, and PI3K-Akt signaling pathway. Huang et al. (2020) found that XCHD significantly inhibits the upregulation of Hsp47, TGF-β1, Col1A1, α-SMA, etc., mediating the HSP47/TGF-β axis to protect the liver. Wang et al. (2023a) found that XCHD significantly alleviated liver fibrosis in a mouse model, improving liver function, reduction liver collagen accumulation, and inhibition of HSC activation. Besides chronic liver diseases, XCHD is also widely used in clinical practice for its regulatory effects on common mental disorders such as depression (Sun et al., 2023).

#### Huangqi decoction

Huangqi decoction (HQD) has been used for the preventing and treating hepatic fibrosis and cirrhosis since ancient times. Zhang et al. (2015) found that HQD significantly downregulated the expression of PDGFrb, PDGFra, PDGFd, PDGFb, THBS1, COL1A1, COL1A2, and COL5A2 in rat liver fibrosis, and downregulated PDGF and TGF-β signaling pathways, thus treating cirrhosis. Liu et al. (2012) found that HQD promotes CD68 expression in liver fibrosis development and protects against liver cell apoptosis. Wang et al. (2021) identified various active components in HQD for liver fibrosis using a pharmacokinetics-based strategy, which was validated in the CCl4-induced mouse liver fibrosis model. Dong et al. (2023) found that HQD adjusted the LCC-C18ORF26-1/miR-663a/TGF-b1/TGF-bRI/p-Smad2 axis to inhibited HSCs activation and proliferation, preventing liver cirrhosis. HQD also alleviates biliary fibrosis and chronic cholestatic liver injury by inhibiting the NF-κB pathway and inducing the Nrf2 pathway in a chronic cholestatic mouse model, preventing liver cirrhosis onset and exacerbation (Li et al., 2019).

#### Dahuang Zhechong pill

The traditional prescription Dahuang Zhechong pill (DHZCP), consisting of rhubarb, native turtle worm, leech, tabanus worm, scutellaria baicalensis, and peach kernel, promotes blood circulation, Gong et al. (2018) found that DHZCP inhibits p-Erk and regulates other factors to inhibit p-p38 by increasing the expression of growth arrest specific 5 (GAS5), alleviating liver fibrosis and preventing cirrhosis. Another study by Gong et al. (2020) found that DHZCP prevents cirrhosis in animal models by inactivating the PI3K/Akt pathway, protecting the liver from damage. Fu et al. (2023) found that DHZCP improves hepatic sinusoids’ capillaries by inhibiting the MK/Itgα signaling pathway, delaying disease progression. Key active molecules affecting this pathway include Liquiritin, Naringenin, Rhein, and PMEG. A systematic review and meta-analysis found that DHZCP reduces the serum biomarkers of liver fibrosis in chronic hepatitis B patients (Wei et al., 2015). Combining Entecavir and DHZCP is an effective intervention for cirrhosis, with an optimal treatment course of about 6 months (Ye et al., 2022). He et al. (2024) recently found that DHZCP effectively regulates the enterohepatic axis, improving intestinal barrier integrity by regulating metabolites and intestinal flora, ultimately inhibiting liver fibrosis progression.

#### Xiayuxue decoction

Xiayuxue decoction (XYXD) came from Jingui Yaolue. Zhang et al. (2020) prepared different fractions including ethyl acetate fraction (EF) from XYXD. It was found that the potential mechanism of EF prevention of liver cirrhosis may be related to the induction of apoptosis of HSCs. Ma et al. (2017) believe that enterogenic endotoxin plays an important role in the further development of liver fibrosis, and experimental studies have confirmed that XYXD treatment can significantly improve CCl4-induced cell death and intestinal inflammation, indicating that XYXD can inhibit the further development of liver fibrosis by improving intestinal epithelial damage. In addition to interfering with the progression of liver cirrhosis, another study (Liu et al., 2015) found that XYXD can also promote the apoptosis of macrophages in rats with renal histopathological damage caused by liver cirrhosis, thus reducing kidney damage in rats. A recent pharmacological study (Deng et al., 2024) also found that XYXD was able to exert significant anti-HCC effects by modulating the interaction between bile acids and intestinal flora, ultimately triggering the immune action of NKT cells against HCC.

#### Yiguanjian decoction

As a more commonly used classical prescription, Yiguanjian (YGJ) decoction is widely used clinically to treat liver disease, which mainly consists of rehmannia, Radix saphora, Angelica, ophiopogon, and wolfberry. Related studies (Tian et al., 2016) found that YGJ was able to increase the expression of MTH1 and Bax, decrease the expression of TNF-α, and significantly improve the degree of hepatic inflammation and fibrosis in mice with chronic hepatitis in a mouse model of hepatic injury, and ultimately was able to inhibit DNA damage in mice with immune liver damage. Relevant researchers (Zhou et al., 2015) found that YGJ and its main component iYGJ can inhibit liver angiogenesis in cirrhotic mice by inhibiting the HIF-1α/VEGF signaling pathway, and finally confirmed that its anti-angiogenesis effect is closely related to improving the liver hypoxic microenvironment. Xu et al. (2018) also established a liver cirrhosis rat model and treated the liver cirrhosis rats with YGJ, and found that YGJ can enhance the liver cirrhosis repair of FLSPC-mediated by regulating the activation state of macrophages, and finally confirmed that YGJ combined with stem cell transplantation is one of the main methods for the treatment of end-stage liver cirrhosis. Through more in-depth studies, Wang et al. (2012) confirmed that YGJ can also inhibit the transfer of bone marrow cells to the liver, further inhibit the differentiation of bone marrow cells, inhibit the proliferation of hepatocytes and progenitor cells in the injured liver, and ultimately play a role in the treatment of liver fibrosis and fundamentally prevent the formation of cirrhosis. Some scholars (Xu et al., 2021) also believe that YGJ can regulate the state of macrophages, and the main anti-cirrhosis effect of YGJ is related to its ability to inhibit the polarization of macrophages M1.

#### Other prescriptions

Kangxian ruangan (KXRG) capsule is a classic homemade prescription. It is composed of seven Chinese medicines, including salvia miltiorrhiza, red peony root, bupleurum, and turtle shell. It has the key role of supplementing the spleen and warming Yang. Clinically, it can significantly reduce or even reverse liver fibrosis and prevent the development of liver cirrhosis. Relevant scholars (Liu et al., 2018) have conducted experimental studies on KXRG and found that it can effectively alleviate liver fibrosis, histopathological changes, and inflammation in rats, and further relieve liver inflammation by maintaining the balance of TNF-α/IL-10, positively regulate the balance of Bcl-XL/Bax or Bcl-2/Bax, and activate damaged liver cells. It can alleviate the progression of nonalcoholic fatty liver disease from liver fibrosis to cirrhosis. As one of the clinical experience prescriptions for the treatment of cirrhosis, KXRG has a remarkable effect, which lays a foundation and provides a direction for the treatment of cirrhosis by modern Chinese medicine in the future.

Chinese medicine decoctions show promise in treating liver cirrhosis by reducing inflammation, improving liver function, and preventing fibrosis. However, studies often have small sample sizes, lack standardized protocols, and offer limited long-term data. Most research is regionally confined to China, requiring broader validation to confirm efficacy and safety globally.

### Chinese patent medicine

#### An-Luo-Hua-Xian

An-Luo-Hua-Xian pill (ALHX) is one of the commonly used Chinese patent medicine for the treatment of liver cirrhosis. As a patented medicine, it has been used in China for more than 15 years. It has the effects of invigorating spleen and nourishing liver, nourishing blood and promoting blood circulation, and softening and dispersing masses. In clinical practice, it is mainly used for chronic hepatitis B and early and middle stage cirrhosis after hepatitis B, and has achieved good curative effect. A clinical study (Liu et al., 2023) of 780 patients found that ALHX combined with Entecal can significantly improve liver fibrosis in patients with chronic hepatitis B and prevent further progression to cirrhosis, so the clinical study to some extent confirmed the positive effect of ALHX in the treatment of liver disease. Xiao and many other researchers (Xiao et al., 2022) conducted a randomized controlled trial that confirmed that chronic hepatitis B patients with F ≤ 2 and alanine aminotransferase (ALT) < 2ULN treated with ALHX could improve the degree of liver fibrosis after 48 weeks. The results of related meta-analysis also confirmed that ALHX and Entecavir had a significant effect on liver fibrosis or cirrhosis (Song et al., 2019). An earlier study (Huang et al., 2009) also found that ALHX was also effective in alleviating liver fibrosis caused by schistosomiasis and further confirmed its excellent hepatoprotective and antifibrotic effects.

#### Fuzhenghuayu

Fuzhenghuayu (FZHY) Decoction is also one of the Chinese patent medicines used to treat cirrhosis clinically, many important components of which are compounds extracted from natural plants. Now, the effect of Fuzhenghuayu (FZHY) decoction has been further proved in clinical studies and experiments. Liu et al. (2019) found that FZHY not only alleviates liver fibrosis and impedes its further progression to cirrhosis, but also improves vascular remodeling in mouse models. Song et al. (2018) recruited 113 subjects, including 63 cirrhosis patients and 50 healthy controls, and applied FZHY to their intervention. The curative effect evaluation showed that the liver stagnation syndrome (LSS) and liver-kidney Yin deficiency syndrome (LKYDS) were effective. The metabolic mechanism may be related to the improvement of the LSS energy supply and the enhancement of the LKYDS detoxification function. Another multi-center clinical study (Liu et al., 2005) found that FZHY can significantly reduce inflammation score and mean inflammatory activity, and has a very good therapeutic effect on chronic hepatitis B-induced liver fibrosis, and the efficacy is better than the Heluoshugan capsule. In addition, Hu et al. (2019) confirmed through animal experiments that FZHY can rapidly upregulate the expression of metabolic enzymes such as Gpt, Adh1, and Hk2, downregulate the expression of Acss2 and Gs, and can also improve rat liver fibrosis and prevent the occurrence of cirrhosis by changing metabolic pathways. Chen et al. (2016) also identified major biomarkers for evaluating the efficacy of FZHY in the treatment of cirrhosis caused by chronic hepatitis B based on transcriptional profiling and miRNA-target network analysis. A miRNA panel with -326, -1182, hsa-miR-18a-5p and -193b-5p were established.

#### Biejia-ruangan

In recent years, Biejia-Ruangan (BR) has been approved as an anti-fibrotic Chinese medicine for the treatment of chronic liver disease and has good efficacy in the prevention and treatment of cirrhosis. A double-blind randomized controlled trial (Ji et al., 2022) has shown that BR combined with entecavir significantly reduces the risk of hepatogenic death and liver cancer in patients with chronic viral hepatitis B and cirrhosis. Another similar study (Rong et al., 2022) also found that in chronic hepatitis B patients with cirrhosis, adding BR to the current standard treatment can help better improve liver fibrosis and alleviate clinical symptoms. Yang et al. (2013) also found in relevant experimental studies that BR can significantly inhibit the progression of liver fibrosis *in vitro* and *in vivo*, and its main mechanism of action may be closely related to the downregulation of fibrosis signal transduction in the TGF-β-Smad pathway. A meta-analysis (Meng et al., 2022) of 26 studies involving 2,717 patients showed that BR significantly improved liver function alleviated the degree of liver fibrosis, and prevented the occurrence of cirrhosis. Interestingly, one study (Luo et al., 2023) also explored the relationship between BR compound and incidence of HCC, but the exact effect and mechanism of action are not fully understood, and more work or scientific studies are still needed to elucidate the clinical effects of BR in the future. To summarize, the efficacy of using Chinese medicine therapies in the treatment of liver cirrhosis is remarkable and has a wide range of application prospects, which is worthy of our further in-depth excavation and exploration.

Chinese patent medicines such as ALHX, FZHY and BR have shown promise in treating liver cirrhosis by improving liver fibrosis, reducing inflammation, and preventing disease progression. However, limitations include small sample sizes, variability in protocols, and a need for larger, rigorous trials to understand their mechanisms and long-term effects. Most studies are limited to China, requiring broader validation. Studies related to the treatment of cirrhosis by Chinese medicine are shown in Table 1. Comparison of drug/medicine selection and efficacy in the treatment of liver cirrhosis between Western or Chinese medicine is shown in Table 2.

**TABLE 1:** Studies related to the treatment of liver cirrhosis with Chinese medicine.

Chinese medicine treatment		Objects	Key findings	References
Chinese medicine decoction	Yinchenhao decoction	BalB/c mice	Yinchenhao decoction regulates the body’s immune and inflammatory responses, reducing the degree of liver inflammation and necrosis	Jiang et al. (2015)
		Network pharmacology, Wistar rats, L02 cells and LX2 cells	Yinchenhao decoction relieves symptoms of liver fibrosis while reducing apoptosis of liver parenchymal cells	Cai et al. (2019)
		SD rats	Yinchenhao decoction inhibits activation and proliferation of HSCs by modulating the TGF-β1/Smad/ERK signaling pathway	Cai et al. (2018)
		Wistar rats	Yinchenhao decoction effectively inhibited α-SMA activation in liver cirrhotic rats	Liu et al. (2008)
	Xiaochaihu decoction	Network pharmacology, Molecular docking	Xiaochaihu decoction can have preventive and therapeutic effects on liver cirrhosis by modulating relevant signaling pathways	Qiang et al. (2022)
		BALB/c mice, Raw 264.7 cells	Xiaochaihu decoction is able to act on HSP47/TGF-βpathway and exerts anti-liver fibrosis effects	Huang et al. (2020)
		Network pharmacology, C57BL/6 mice	Xiaochaihu decoction inhibits HSC activation, improves liver function, and also reduces hepatic collagen accumulation	Wang et al. (2023a)
	Huangqi decoction	Network pharmacology, Wistar rats	Huangqi decoction regulates TGF-β and PDGF signaling pathways and exerts positive therapeutic effects on liver fibrosis	Zhang et al. (2015)
		Wistar rats	Huangqi decoction promotes CD68 expression and protects hepatocytes from apoptosis	Liu et al. (2012)
		C57BL/6 mice, LX-2 cells	Various active ingredients of Huangqi decoction can have positive therapeutic effects on mice with liver fibrosis	Wang et al. (2021)
		LX2 and LO2 cells	Huangqi decoction can inhibit the activation and proliferation of HSCs and prevent the further development of cirrhosis	Dong et al. (2023)
	Dahuang Zhechong pill	SD rats, HSC-T6 cells	Dahuang Zhechong pills inhibit the expression of p-Erk and p-p38, which ultimately exerts an anti-hepatic fibrosis effect	Gong et al. (2018)
		SD rats, HSC-T6 cells	Dahuang Zhechong pills can promote the inactivation of the PI3K/Akt pathway and exert a hepatoprotective effect	Gong et al. (2020)
		SD rats, Molecular docking	Dahuang Zhechong pills inhibited the MK/Itgα signaling pathway and improved hepatic sinusoidal capillarization	Fu et al. (2023)
	Xiayuxue decoction	C57BL/6 mice, JS1 and LX2 cells	Xiayuxue decoction exerts therapeutic effects on liver cirrhosis by inducing HSC apoptosis	Zhang et al. (2020)
		C57BL/6 mice	Xiayuxue decoction ameliorates intestinal epithelial injury to alleviate hepatic fibrosis in mice	Ma et al. (2017)
	Yiguanjian decoction	C57BL/6 mice	Yiguanjian decoction improves hepatic hypoxic microenvironment and anti-angiogenic effect and inhibits liver cirrhosis progression in mice	Zhou et al. (2015)
		Wistar rats, WB-F344 cells	Yiguanjian decoction can regulate the activation status of macrophages, thus promoting the recovery of liver cirrhosis	Xu et al. (2018)
		ICR mice	Yiguanjian decoction inhibits the metastasis of bone marrow cells to the liver and also inhibits the differentiation of bone marrow cells, thus preventing cirrhosis from occurring	Wang et al. (2012)
		Wistar rats, RAW264.7 cells	Yiguanjian decoction inhibits macrophage M1 polarization and thus exerts anti-cirrhotic effects	Xu et al. (2021)
	Kangxian ruangan capsule	Wistar rats	Kangxian ruangan capsule can activate damaged liver cells and alleviate liver inflammation, easing the further development of liver cirrhosis	Liu et al. (2018)
Chinese patent medicine	An-Luo-Hua-Xian	Clinical trial	Combination therapy with entecavir and An-Lo-Hua-Xian pill significantly improves liver fibrosis in patients with chronic hepatitis B	Liu et al. (2023)
		Clinical trial	Chronic hepatitis B patients with significant remission of liver fibrosis after 48 weeks of An-Luo-Hua-Xian pill treatment	Xiao et al. (2022)
	Fuzhenghuayu	C57BL/6 mice, Human hepatic sinusoidal endothelial cells, Human umbilical vein endothelial cells	Fuzhenghuayu decoction improves vascular remodeling and inhibits further development of liver cirrhosis	Liu et al. (2019)
		Clinical trial	Fuzhenghuayu formula is able to treat patients with different types of cirrhosis by improving energy supply and enhancing detoxification	Song et al. (2018)
		Clinical trial	Fuzhenghuayu capsule has significant anti-inflammatory activity and can alleviate liver fibrosis caused by chronic hepatitis B without adverse effects	Liu et al. (2005)
		Wistar rats	Fuzhenghuayu formula ameliorates liver fibrosis in rats by modulating gene expression of related metabolic enzymes and altering metabolic pathways	Hu et al. (2019)
		Clinical trial	Study identifies key biomarkers for evaluating the efficacy of Fuzhenghuayu formula in the treatment of chronic hepatitis B cirrhosis	Chen et al. (2016)
	Biejia-Ruangan	Clinical trial	Entecavir in combination with Biejia-Ruangan compound reduces the risk of HCC and hepatogenous death in patients with hepatitis B combined with liver cirrhosis	Ji et al. (2022)
		Clinical trial	Biejia-Ruangan tablet combined with modern medicine can better improve liver fibrosis and relieve clinical symptoms	Rong et al. (2022)
		Wistar rats, HSC-LX-2 cells	Fufang Biejia Ruangan pills significantly inhibited the progression of liver fibrosis in vivo and *in vitro* experiments	Yang et al. (2013)
		Systematic review, Meta-analysis	Biejia Ruangan tablets significantly reduced liver fibrosis and improved liver function without serious adverse effects	Meng et al. (2022)

**TABLE 2:** Drug/medicine selection and efficacy in the treatment of cirrhosis by Western or Chinese medicine.

Drug/medicine category	Name	Effects	References
Antiviral drugs	Entecavir, tenofovirlamivudine; adefovir dipivoxyl; sorfosbuvir	Inhibit viral replication, reduce liver damage	Marcellin et al. (2013), Morgan et al. (2010), Zhang et al. (2017), Jagst et al. (2024)
Hepatoprotective and anti-inflammatory drugs	Silymarin, glycyrrhizic acid preparations, polyene phosphatidylcholine, adenosine methionine, dicycloalcohol; reduced glutathione, UDCA	Inhibit liver inflammation, protect liver cells, promote liver cell regeneration	Damiris et al. (2020), Guo and Lu (2020)
Antifibrotic drugs	Losartan, hydronidone	Inhibit liver fibrosis progression	Gu et al. (2023), Sun et al. (2024)
Diuretics	Furosemide, spironolactone, tovaptan, terlipressin	Reduce ascites and edema	Malick et al. (2023), Pratama et al. (2024), Ding et al. (2024)
Anti-hepatic encephalopathy drugs	Rifaximin, lactulose/lactitol	Reduces ammonia production and maintains intestinal cleanliness	Rajpurohit et al. (2022)
Chinese medicine decoctions	Yinchenhao, Xiaochaihu, Huangqi, Dahuang Zhechong, Xiayuxue, Yiguanjian, Kangxian ruangan	Reduce inflammation, improve liver function, prevent liver fibrosis	Jiang et al. (2015), Qiang et al. (2022), Zhang et al. (2015), Gong et al. (2018), Zhang et al. (2020), Tian et al. (2016), Liu et al. (2018)
Chinese patent medicines	An-Luo-Hua-Xian, Fuzhenghuayu, Biejia-ruangan	Improves liver fibrosis, reduces inflammation, prevents disease progression	Liu et al. (2023), Liu et al. (2019), Ji et al. (2022)

### Acupuncture treatment

The ancient practice of acupuncture is gaining popularity around the world. More than 30 years ago, the World Health Organization began recommending acupuncture therapy. Another decade later, the NIH in the United States also reached a consensus on the effectiveness of acupuncture therapy. As an effective non-pharmacological treatment to reduce the symptoms of cirrhosis, it can be used as an alternative or complementary treatment to improve the efficacy of medication, and compared with medication, acupuncture has the advantages of low cost, low side effects and quick results. Recent studies (Wang et al., 2023b) have found that acupuncture also has a significant immunomodulatory effect, playing a key role in regulating the innate immune system, adaptive immune system, etc. It is effective in regulating the body’s physiological functions through the stimulation of specific body points. Acupuncture therapy is gradually entering the mainstream medical system. In general, “the position of acupuncture feeling, the position of disease response, and the location of therapeutic effect” are the three major functions of acupuncture points, and they are also the basic points for understanding the principle of acupuncture action, which is worthy of in-depth study by our later generations. Nowadays, acupuncture not only has a good regulation effect on the complications of cirrhosis but also has a good effect on the treatment and prevention of cirrhosis. Zhang et al. (2012a) found through the experimental study that acupuncture can significantly improve the therapeutic effect of curcumin on CCl4-induced liver fibrosis in rats, and effectively reduce the levels of serum aspartate aminotransferase (AST), ALT, and hyaluronic acid. Other researchers (Zhang et al., 2012b) have also found that the combination of acupuncture and curcumin in the treatment of liver fibrosis significantly enhances the effect of curcumin at the molecular level, the main mechanism of action is that it stimulates the degradation of the ECM in the fibrotic liver and the destruction of the PDGF-βR/ERK pathway. Other relevant literature (Zhou et al., 2012) has found that acupuncture has potential advantages in the prevention of cirrhosis because acupuncture can better reduce the body’s qi and blood stasis and enhance the body’s immunity. The results of the meta-analysis (Qi et al., 2020) also show that acupuncture is an important treatment choice for patients with cirrhosis as a complementary therapy.

Acupuncture has gained global acceptance as a low-cost, effective treatment for cirrhosis, endorsed by the WHO and NIH. It offers immunomodulatory benefits and enhances treatments like curcumin for liver fibrosis. However, research is often limited by small sample sizes, lack of rigorous controls, unclear long-term effects, and variability in techniques, necessitating broader validation.

### Combination of Chinese and Western medicine in the treatment of liver cirrhosis

At present, the treatment of liver cirrhosis with integrated traditional Chinese and Western medicine is widely popular in China, supported by substantial related research. Regarding ascites in cirrhosis, a recent experimental study found that combining YCHD with spironolactone can prevent sodium-potassium imbalance, providing significant guidance for managing ascites in liver cirrhosis (Hsueh and Tsai, 2023). For chronic hepatitis B cirrhosis, a clinical study involving 116 patients demonstrated that the group treated with both Chinese medicine and Western medicine showed superior results compared to the control group. Specifically, the combined treatment group exhibited better outcomes in terms of ALT, AST, total bilirubin, alpha-fetoprotein levels, coagulation function indicators, liver elasticity values, and overall efficacy. Additionally, the incidence of complications such as spontaneous bacterial peritonitis, hepatic encephalopathy, hepatorenal syndrome, gastrointestinal bleeding, and electrolyte imbalance was significantly lower in the combined treatment group. The authors concluded that integrating Chinese medicine and Western medicine in the treatment of cirrhosis can effectively reduce complications, improve clinical symptoms, and enhance clinical efficacy, making it worthy of further research and clinical application (You et al., 2023). In another prospective randomized controlled trial on hepatitis B cirrhosis, it was found that the antiviral entecavir combined with Fuzheng Huayu tablets improved the biochemical response rate and showed trends towards improving HBeAg seroconversion and liver fibrosis rates, compared to the control group (entecavir plus a placebo). Importantly, no serious adverse events related to the study treatment were observed during the trial (Cheng et al., 2022). Regarding primary biliary cholangitis, Chinese scholars conducted a systematic review and meta-analysis of randomized controlled trials on the combined treatment of Chinese medicine and UDCA. The findings confirmed that Chinese medicine adjunctive therapy with UDCA had significant advantages over UDCA alone. Specifically, Guishao Huoxue (GSHX) decoction combined with UDCA was beneficial for the clinical efficacy rate, while ALHX pill combined with UDCA had more comprehensive effects on liver function indicators such as alkaline phosphatase and TBil (Chen et al., 2020).

These findings collectively highlight the potential benefits of integrating traditional Chinese and Western medicine in the treatment of liver cirrhosis, warranting further research and clinical promotion. However, current research on this integrated approach has several limitations. These include limited sample sizes, potential biases due to study design issues, unclear long-term effects, inadequate reporting of adverse effects, insufficient mechanistic research, regional limitations with most studies conducted in China, and variability in treatment methods and dosages, which affect the reproducibility and comparability of results.

Future drug treatments should focus on multi-target combination therapies to enhance efficacy and reduce side effects. The development of new antifibrotic drugs, such as RNA interference and gene editing technologies (Rajput et al., 2021), will be a key research focus. Further high-quality evidence-based studies are required to validate the efficacy and safety of integrating Chinese and Western medicine. Personalised treatment, guided by genomics and other omics technologies, will tailor therapies to individual patients, improving outcomes and reducing adverse effects (Park et al., 2022; Yang et al., 2023). The integration of nanotechnology and biomaterials will facilitate the development of more effective drug delivery systems, ensuring a more precise targeting of diseased areas. Immunomodulatory drugs and a comprehensive approach to lifestyle management will also play a pivotal role in future strategies, collectively improving patient prognosis. By integrating innovation, personalisation, and advanced delivery systems, liver cirrhosis treatment will continue to evolve and optimise.

## Conclusions and perspective

After continuous research and exploration, research related to liver cirrhosis has achieved certain results. Modern medicine mainly believes that viral hepatitis, non-alcoholic fatty liver disease, and other related etiologies promote the occurrence and development of cirrhosis, while the pathogenesis of cirrhosis is closely related to the activation of HSC and the regulation of LSECs. At present, the treatment of liver cirrhosis in Western medicine is mostly based on etiologic treatment, anti-inflammatory and antifibrotic treatment, as well as prevention and treatment of cirrhosis complications, which cannot be completely cured. However, Chinese medicine is widely used in the treatment of liver fibrosis, cirrhosis, and its related complications, based on the characteristics of multi-component, multi-target, multi-pathway, and the combination of holistic and local treatment of cirrhosis, which has achieved good therapeutic effects in the clinic and greatly enriched the prevention and treatment means of chronic liver diseases in the clinic (Li 2020; Pun et al., 2024). Chinese medicine is a treasure of Chinese traditional culture. In-depth excavation of the key target links of traditional Chinese medicine interventional therapies, such as Chinese medicine monomer, Chinese medicine decoction, Chinese patent medicine as well as acupuncture, closely integrates the theoretical thinking of Chinese medicine with modern medicine and provides brand-new ideas and better therapeutic strategies for the treatment of liver cirrhosis and other chronic liver diseases.

The integration of Western and Chinese Medicine in the treatment of cirrhosis is a complex landscape. Current treatments in Western medicine, while effective in managing symptoms and complications, often fail to completely halt disease progression. Chinese medicine offers a holistic approach with multi-target strategies, including herbal decoctions and acupuncture, which have shown promise in reversing liver fibrosis and improving overall liver function. However, there is a grey area where the lack of standardised treatment protocols and comprehensive clinical trials creates challenges in validating the efficacy of Chinese medicine within the framework of Western medicine. To fill these gaps, rigorous scientific studies are needed, including high-quality multicentre clinical trials and advanced pharmacological research to elucidate the mechanisms behind Chinese medicine therapies. Future developments should focus on creating integrative treatment protocols that leverage the strengths of both medical systems, ultimately improving patient outcomes and advancing the understanding of cirrhosis management.

However, despite the progress made, several limitations persist in current research. Firstly, there is a dearth of animal models that effectively integrate Chinese medicine theories, basic experiments, and clinical observations. Future research endeavors should thus strive for innovative approaches that align closely with the principles of Chinese medicine. Secondly, existing Chinese medicine research on cirrhosis predominantly relies on animal experimentation, resulting in a limited evidence chain and insufficiently detailed mechanistic insights. Future efforts should aim to deepen our understanding of the mechanisms underlying Chinese medicine interventions for cirrhosis, employing diverse research methodologies to strengthen the evidence base. Moving forward, there is a pressing need to undertake high-quality, multicenter, large-scale, and standardized clinical evidence-based research, integrating it with collaborative basic experiments. This approach aims to facilitate the translation of Chinese medicine-related research outcomes into clinical practice. Furthermore, it remains imperative to leverage the distinctive advantages of Chinese medicine in preventing and treating cirrhosis and other chronic liver diseases. Harnessing the creativity and distinctiveness of Chinese medicine, deeper exploration into the molecular mechanisms underlying Chinese medicine’s therapeutic effects is warranted. This exploration should lead to the development of novel proprietary Chinese medicines and their judicious combination with Western medicines, thereby enhancing the quality of diagnosis and treatment for cirrhosis patients. Simultaneously, it is essential to perpetuate and elevate the heritage of Chinese medicine culture. It is my belief that in the future, we will achieve groundbreaking discoveries akin to Artemisinin and make significant contributions to the field of human medicine.
